# Bacterial chemotaxis towards polysaccharide pectin by pectin-binding protein

**DOI:** 10.1038/s41598-020-60274-1

**Published:** 2020-03-04

**Authors:** Hidenori Konishi, Mamoru Hio, Masahiro Kobayashi, Ryuichi Takase, Wataru Hashimoto

**Affiliations:** 1 Laboratory of Basic and Applied Molecular Biotechnology, Division of Food Science and Biotechnology, Graduate School of Agriculture, Kyoto University, Uji, Kyoto 611-0011 Japan; 2 Laboratory of Basic and Applied Molecular Biotechnology, Department of Food Science and Biotechnology, Faculty of Agriculture, Kyoto University, Uji, Kyoto 611-0011 Japan

**Keywords:** Polysaccharides, Bacteriology

## Abstract

As opposed to typical bacteria exhibiting chemotaxis towards low-molecular-weight substances, such as amino acids and mono/oligosaccharides, gram-negative *Sphingomonas* sp. strain A1 shows chemotaxis towards alginate and pectin polysaccharides. To identify the mechanism of chemotaxis towards macromolecules, a genomic fragment was isolated from the wild-type strain A1 through complementation with the mutant strain A1-M5 lacking chemotaxis towards pectin. This fragment contained several genes including *sph1118*. Through whole-genome sequencing of strain A1-M5, *sph1118* was found to harbour a mutation. In fact, *sph1118* disruptant lost chemotaxis towards pectin, and this deficiency was recovered by complementation with wild-type *sph1118*. Interestingly, the gene disruptant also exhibited decreased pectin assimilation. Furthermore, the gene product SPH1118 was expressed in recombinant *E. coli* cells, purified and characterised. Differential scanning fluorimetry and UV absorption spectroscopy revealed that SPH1118 specifically binds to pectin with a dissociation constant of 8.5 μM. Using binding assay and primary structure analysis, SPH1118 was predicted to be a periplasmic pectin-binding protein associated with an ATP-binding cassette transporter. This is the first report on the identification and characterisation of a protein triggering chemotaxis towards the macromolecule pectin as well as its assimilation.

## Introduction

Bacterial cells move to preferred conditions or leave adverse ones using their flagella, depending on the concentration gradient of chemotactic substances in the environment; this is referred to as chemotaxis^1^. The flagellum comprises a basal body, hook and filament. In many cases, except for some bacteria such as *Rhodobacter* sp., when the flagellum turns counter-clockwise, the bacterium generally moves forward, whereas when the flagellum turns clockwise, the bacterium changes direction^2,3^. The flagellum of *Rhodobacter* sp. rotates clockwise when moving straight and stops rotating when changing the direction^4^. Methyl-accepting chemotaxis protein (MCP) is a well-known receptor of chemotactic substances. MCP is a membrane protein comprising three domains: N-terminal ligand-binding domain, membrane-spanning domain and C-terminal signalling domain^5^. Low-molecular-weight substances act as ligands for MCPs, and MCPs in *Escherichia coli* have been well studied. *E. coli* exhibits four types of MCPs which recognise various low-molecular-weight substances. Tar receptor binds to aspartic acid and maltose (more specifically to maltose-bound proteins, as described later); Tsr receptor to serine and leucine; Trg receptor to ribose and galactose and Tap receptor to dipeptide and pyrimidine^6–8^.

As opposed to these bacteria, the motile strain A1-M5 has been isolated from the non-motile wild-type gram-negative *Sphingomonas* sp. strain A1 after repetitive subculture on a semisolid plate^9^. Strain A1-M5 exhibits chemotaxis towards the high-molecular-weight substance alginate polysaccharide but not towards pectin polysaccharide^10^, although both alginate and pectin are well assimilated by strain A1 cells. Alginate, produced by brown seaweed and certain bacteria, is an acidic polysaccharide composed of β-d-mannuronate and its C5 epimer α-l-guluronate^11^. Pectin contained in plant cell walls constitutes three regions: polygalacturonan (PG), rhamnogalacturonan type I (RG-I) and rhamnogalacturonan type II (RG-II)^12^. PG is a homopolymer composed of d-galacturonate (GalUA). RG-I is composed of alternating l-rhamnose and GalUA as the main chain and arabinans, galactans and arabinogalactans as side chains. RG-II contains PG as the main chain and branched sugar side chains. Both alginate and pectin are thus categorised as acidic polysaccharides due to their high levels of uronic acid residues.

Strain A1 cells assimilate alginate in a unique way (Fig. S1). Alginate is directly incorporated as a polysaccharide into the cytoplasm through the mouth-like pit and ATP-binding cassette (ABC) transporter on the cell surface^13,14^. This mechanism has been focused on as novel macromolecular import machinery that acts independent of extracellular macromolecule-degrading enzymes. Periplasmic alginate-binding proteins (AlgQ1 and AlgQ2) show an affinity towards alginates with a dissociation constant (*K*_d_) of 0.2 µM^15^ and deliver the polysaccharide to the ABC transporter. Alginate ABC transporter of strain A1 comprises heterodimeric AlgM1–AlgM2 as a cytoplasmic membrane-spanning protein and homodimeric AlgS–AlgS as an ATPase^16^. Determination of crystal structure of the ABC transporter in the complex with alginate-binding protein has offered structural insights into macromolecule alginate import^17^. Alginate transported across the cytoplasmic membrane by the ABC transporter is finally degraded to monosaccharides in the cytoplasm by endo- and exo-type alginate lyases (A1-I, II, III and IV)^18,19^.

The gram-negative bacterium *Dickeya dadantii* (formerly known as *Erwinia chrysanthemi*) exhibits chemotaxis towards pectin oligosaccharides^20^. In *D. dadantii*, pectin oligosaccharide is incorporated into the periplasm and bound to a periplasmic solute-binding protein (TogB)^21^. TogB delivers pectin oligosaccharide to an ABC transporter (TogMNA_2_). Alternatively, TogB also functions as a receptor for chemotaxis^20^, as is seen in the import of maltose by *E. coli* showing chemotaxis towards maltose^22^. Although maltose is incorporated into the cytoplasm by a maltose-binding protein (MalE or MBP)-dependent ABC transporter (MalFGK_2_)^23^, the maltose-bound form of MalE also acts as a receptor for chemotaxis towards maltose^24^. Maltose-bound MalE interacts with the periplasmic domain of Tar receptor to transmit chemotactic signals^25,26^. However, to the best of our knowledge, there have been no reports on bacterial chemotaxis towards pectin polysaccharides.

In this study, another motile strain, A1-MP, was obtained from strain A1 and found to exhibit chemotaxis towards both alginate and pectin. Thus, strain A1-M5 cells may lack in pectin recognition for chemotaxis. Strain A1 cells produce a periplasmic alginate-binding protein, suggesting the presence of a pectin-binding protein as a receptor for chemotaxis towards pectin. This article describes the molecular identification and characterisation of a pectin-binding protein as a receptor for chemotaxis towards pectin polysaccharide through genome-wide sequencing, gene complement assay and *in vitro* protein assay.

## Results

### Bacterial chemotaxis towards pectin and alginate

**Figure 1:**
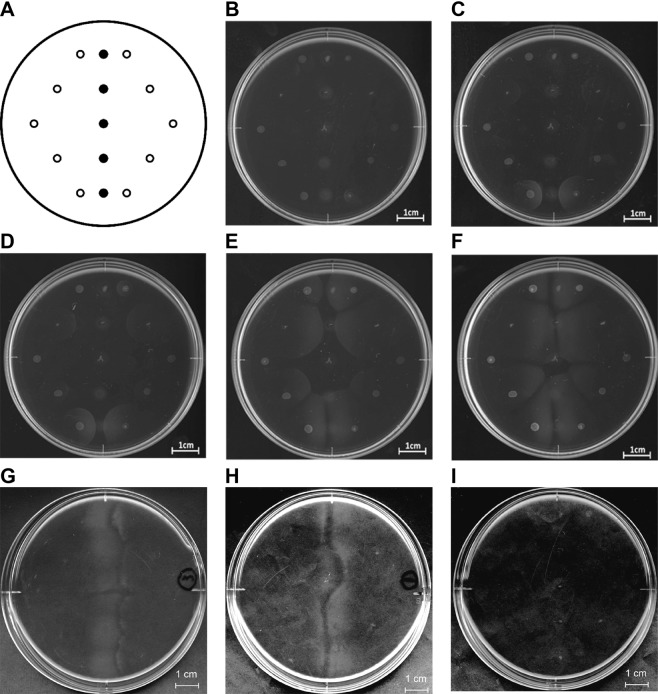
Bacterial chemotaxis towards alginate and pectin. (**A**) Chemotaxis assay plate. Closed circle, spot site for polysaccharide solution; open circle, spot site for bacterial cell suspension. Unless otherwise specified, images of the chemotaxis assay plate were obtained after incubation of the bacterial cells with the polysaccharide for 5 days. (**B**–**F**) Time course of chemotaxis assay. Strain A1-MP cells were spotted with pectin and incubated for 5 days. Images were periodically obtained at 1 (**B**), 2 (**C**), 3 (**D**), 4 (**E**) and 5 (**F**) days. On the 2- or 3-day plate, strain A1-MP cells spotted at the nearest pectin began to move towards the polysaccharide. (**G**) Chemotaxis of strain A1-MP cells towards alginate. (**H**,**I**) Chemotaxis of strain A1-M5 cells towards alginate (**H**) but not towards pectin (**I**). Except for plates (**A**,**I**), the grey area in the middle of the plate stems from accumulating cells. Each plate assay was replicated three times independently. The profile of each plate is not an image cropped from different parts of the same plate or from different plates.

A vast majority of bacterial cells with flagella are well known to be motile on soft agar plates^27^. Cells of the motile strain A1-MP were isolated from cells of the non-motile strain A1 after repetitive subculture on a semisolid plate with 0.5% agar in a similar way used to obtain cells of strain A1-M5^9^ and were subsequently subjected to chemotaxis assay (Fig. 1A). Strain A1-MP cells periodically moved to pectin in a ligand-concentration-dependent manner (Fig. 1B–F); these cells also showed chemotaxis towards alginate (Fig. 1G), indicating that strain A1 cells show innate potential for chemotaxis towards pectin and alginate. In contrast, strain A1-M5 cells moved to alginate (Fig. 1H) but not to pectin as a ligand (Fig. 1I), as described previously^10^. Although no artificial mutagenesis was performed during isolation^9^, these strain A1-M5 cells may spontaneously exhibit deficiency in pectin recognition for exhibiting chemotaxis towards this polysaccharide.

To identify the gene responsible for deficiency of chemotaxis towards pectin in strain A1-M5, whole-genome sequence was determined and compared with that of wild-type strain A1. Surprisingly, 297 mutations were identified in strain A1-M5 genome (Table S1), although the mutation rate relative to the overall sequence was <0.007%. These mutations in the strain A1-M5 genome occurred in 104 genes (Table 1). Thus, another approach to identify the gene crucial in pectin recognition for chemotaxis was employed due to the unexpectedly large number of mutant genes in strain A1-M5.

**Table 1:** Genes with mutation in strain A1-M5.

*sph1*	*sph1117*	*sph1993*	*sph3073*
*sph4*	*sph1118*	*sph2070-sph2071*	*sph3103*
*sph11-sph12*	*sph1119*	*sph2090*	*sph3161-sph3162*
*sph69*	*sph1128*	*sph2102*	*sph3165*
*sph76*	*sph1129*	*sph2147-sph2148*	*sph3228*
*sph163-sph164*	*sph1185-sph1186*	*sph2309-sph2310*	*sph3277*
*sph358*	*sph1197-sph1198*	*sph2450*	*sph3278*
*sph407*	*sph1226*	*sph2453*	*sph3319*
*sph473*	*sph1287-sph1288*	*sph2468*	*sph3320*
*sph508*	*sph1295-sph1296*	*sph2534*	*sph3370*
*sph511*	*sph1333*	*sph2572-sph2573*	*sph3447-sph3448*
*sph512*	*sph1396*	*sph2634*	*sph3557*
*sph515-sph516*	*sph1397*	*sph2634*	*sph3575*
*sph574*	*sph1419*	*sph2647*	*sph3600*
*sph575*	*sph1452-sph1453*	*sph2733*	*sph3743*
*sph587-sph588*	*sph1513-sph1514*	*sph2762*	*sph3744*
*sph598-sph599*	*sph1586*	*sph2763*	*sph3813*
*sph770*	*sph1588*	*sph2851-sph2852*	*sph3881*
*sph893*	*sph1877-sph1878*	*sph2852*	*sph3996*
*sph1073*	*sph1919*	*sph2889-sph2890*	
*sph1103-sph1104*	*sph1941-sph1942*	*sph3059*	

### Complementation of strain A1-M5 lacking chemotaxis towards pectin

**Figure 2:**
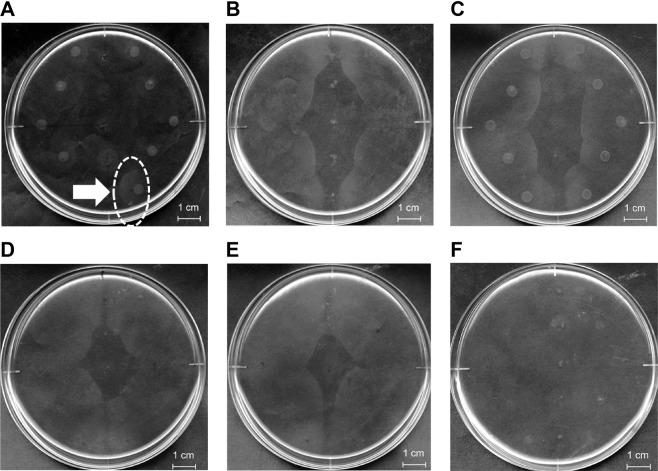
Expression of chemotaxis towards pectin by gene complementation in strain A1-M5 cells. (**A**) Chemotaxis of strain A1-M5 cells complemented by genomic fragments from the wild-type strain A1 cells. (**B**). Chemotaxis of the isolated genomic fragment-complemented strain A1-M5 cells towards pectin. (**C**) Chemotaxis of strain A1-M5 cells transformed with pKS13-pectin towards pectin. (**D**–**F**) Chemotaxis assay of specific gene-complemented strain A1-M5 cells. Chemotaxis towards pectin was observed in *sph1117-sph1118*-complemented strain A1-M5 cells (**D**) and *sph1119*-complemented strain A1-M5 cells (**E**) but not in the *sph2733*-complemented strain A1-M5 cells (**F**). Except for the plate (**F**), the grey area in the middle of the plate stems from accumulating cells. Each plate assay was replicated three times independently. The profile of each plate is not an image cropped from different parts of the same plate or from different plates.

Conversion of strain A1-M5 to a strain A1-MP-like form was attempted by a gene complementation assay using the genome library of strain A1^16^. Strain A1-M5 cells were transformed with pKS13 plasmids including genomic fragments of strain A1 through triparental mating. The resultant transformants of strain A1-M5 cells were subjected to chemotaxis assay using pectin as a ligand. Specifically, transformed strain A1-M5 cells were spotted on a semisolid assay plate, with pectin spotted at the centre. After incubation at 30 °C for 5 days, some cells began to move towards pectin (Fig. 2A, white dotted circle). These motile cells were harvested and streaked on an alginate minimal medium plate containing tetracycline for isolating transformants. Five transformants were isolated from the plate and subjected to pectin chemotaxis assay. All the isolated transformants showed chemotaxis towards pectin (Fig. 2B). To confirm that the introduction of plasmid harboured by the transformants conferred strain A1-M5 cells with the ability to exhibit chemotaxis towards pectin, the plasmid was isolated from each transformant and introduced again into strain A1-M5 cells through triparental mating. Finally, a plasmid designated pKS13-pectin was found to be crucial for the expression of chemotaxis towards pectin in strain A1-M5 cells (Fig. 2C).

### Role of SPH1118 in chemotaxis towards pectin

DNA sequence of strain A1 genomic fragments inserted into the pKS13-pectin plasmid was determined by primer walking using 19 primers (pKS_F1 to f-3135268~ in Table S2). Two genomic fragments of strain A1 were finally found to be included in pKS13-pectin: a genomic fragment (13 kb) at sites 1277036–1289921 containing 9 genes (*sph1111*–*sph1119*) and another (14 kb) at 3122401–3136604 containing 13 genes (*sph2724*–*shp2736*). During construction of the genomic DNA library of strain A1^16^, these two genomic fragments digested with *Sau*3AI appeared to be ligated with *Bam*HI-treated pKS13.

**Figure 3:**
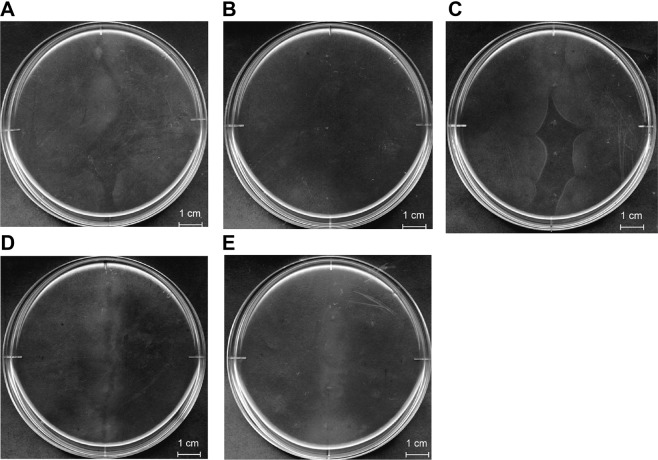
Chemotaxis assay of strains *Δsph1118* and *Δsph1119*. Chemotaxis of strain *Δsph1118* cells towards alginate (**A**) but not towards pectin (**B**). Chemotaxis of *sph1118*-complemented strain *Δsph1118* cells towards pectin (**C**). Chemotaxis of strain *Δsph1119* cells towards alginate (**D**) and pectin (**E**). Except for the plate (**B**), the grey area in the middle of the plate stems from accumulating cells. Each plate assay was replicated three times independently. The profile of each plate is not an image cropped from different parts of the same plate or from different plates.

Among the 22 genes, *sph1117*, *sph1118*, *sph1119* and *sph2733* corresponded to mutated genes in strain A1-M5 as opposed to those in the wild-type strain A1 (Table 1), suggesting that mutations in one or more of these genes conferred strain A1-M5 cells with the ability to exhibit chemotaxis towards pectin. To confirm this hypothesis, strain A1-M5 cells were complemented with each of the three wild-type strain A1 fragments (*sph1117*-*sph1118*, *sph1119* and *sph2733*) through triparental mating, following which the resultant complemented strain A1-M5 cells were subjected to chemotaxis assay. Chemotaxis towards pectin was exhibited by strain A1-M5 cells complemented by *sph1117*-*sph1118* (Fig. 2D) and *sph1119* (Fig. 2E) but not by *sph2733* (Fig. 2F). Because *sph1117* is extremely short (303 bp), this gene was considered an unimportant fragmented form. Hence, the roles of *sph1118* and *sph1119* in chemotaxis towards pectin were investigated through constructing strain A1-MP gene disruptants (strains *Δsph1118* and *Δsph1119*) by knocking out each gene by insertion of a kanamycin-resistant gene cassette (Km^r^). Strain *Δsph1118* cells showed chemotaxis towards alginate (Fig. 3A) but not towards pectin (Fig. 3B), indicating that strain *Δsph1118* cells lost the chemotactic ability towards pectin. In contrast, chemotaxis towards both alginate (Fig. 3D) and pectin (Fig. 3E) was still observed in strain *Δsph1119* cells, similar to that in strain A1-MP. Furthermore, strain *Δsph1118* cells were transformed with the wild-type *sph1118*, and the resultant transformants were subjected to chemotaxis assay. *sph1118*-complemented strain *Δsph1118* cells apparently recovered their chemotactic ability towards pectin (Fig. 3C). These results demonstrate that *sph1118* is crucial for chemotaxis towards pectin.

Pectin assimilation by these strain A1 derivatives was also investigated (Fig. S2). In contrast to strain A1-MP cells (Fig. S2 circle), strain *Δsph1118* (Fig. S2 cross) and strain A1-M5 (Fig. S2 rhombus) cells showed decreased growth in a pectin minimal medium. The growth of *sph1118*-complemented strain *Δsph1118* cells (Fig. S2 square) in the pectin minimal medium was restored to same as that of strain A1-MP cells, indicating that *sph1118* is involved in pectin assimilation as well as polysaccharide chemotaxis. However, these results suggested that *sph1118* disruption diminishes pectin assimilation; therefore, no chemotaxis towards pectin was observed in strain A1-M5 and *Δsph1118* cells. Thus, pectin assimilation by *sph1117*-*sph1118-*complemented strain A1-M5 cells was examined; the complemented cells showed decreased growth in pectin minimal medium (Fig. S2 plus), although their growth rate was comparable to that of strain *Δsph1118* cells. Because *sph1117*-*sph1118*-complemented strain A1-M5 cells showed chemotaxis towards pectin (Fig. 2D), their decreased growth on pectin had little effect on chemotaxis towards pectin. *sph1118*-complemented strain *Δsph1118* cells recovered their ability to assimilate pectin, while little increase in growth of *sph1117*-*sph1118*-complemented strain A1-M5 cells on pectin was observed. These results suggest that strain A1-M5 cells harbour another deleterious mutations related to pectin assimilation in addition to those in *sph1118*. Taken together, SPH1118, the *sph1118*-encoded protein, is essential for chemotaxis towards pectin and its assimilation.

### SPH1118 binding to pectin

In terms of the primary structure, SPH1118 is similar to the periplasmic solute-binding proteins associating with ABC transporters, suggesting that SPH1118 binds to pectin. Thus, to investigate binding of SPH1118 (~70 kDa) to pectin, recombinant SPH1118 with a His tag at the C-terminus was expressed in *E. coli* cells and purified to homogeneity. Purified SPH1118 was eluted as a single peak by gel filtration column chromatography (Fig. S3A), and the purity was also confirmed by sodium dodecyl sulphate–polyacrylamide gel electrophoresis (SDS–PAGE) (Fig. S3B).

**Figure 4:**
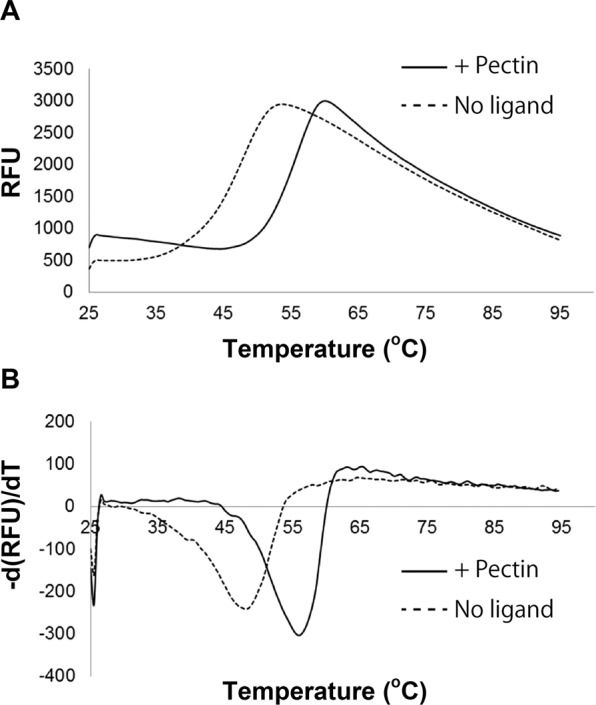
Binding assay between SPH1118 and pectin using DSF. (**A**) Fluorescence profile of SPH1118 in the absence (dotted line) and presence (solid line) of pectin at 5 µM. (**B**) The negative derivative curve plot obtained from the fluorescence profile (**A**). Relative minimal values of SPH1118 in the absence (dotted line) and presence (solid line) of pectin were determined as *T*_m_ values of 48.0 °C and 56.6 °C, respectively.

Differential scanning fluorimetry (DSF) was used to investigate the interaction between SPH1118 and pectin. Generally, ligand-bound proteins are more stable than ligand-free forms^28^. Thermal stability of SPH1118 in the absence or presence of pectin was thus analysed. Fluorescence profile of SPH1118 in the presence of pectin was shifted to a higher temperature than that in the absence of this polysaccharide, indicating that SPH1118 was converted to a stable form in the presence of pectin (Fig. 4A). Melting temperature (*T*_m_) can be calculated as a transition midpoint of the fluorescence profile^28^. The *T*_m_ values of SPH1118 in the absence and presence of pectin were 48.0 °C and 56.6 °C, respectively (Fig. 4B), suggesting that SPH1118 binds to pectin and that the pectin-bound SPH1118 is more stable than its pectin-free form.

**Figure 5:**
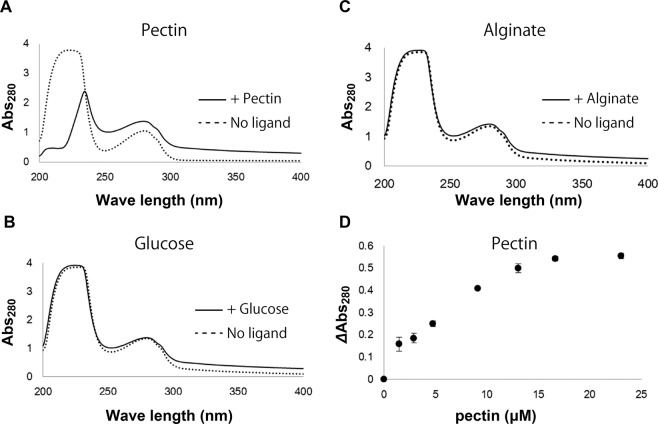
Binding assay of SPH1118 with pectin and pectin components using UV absorption spectroscopy. The UV spectrum of SPH1118 was measured in the absence (dotted line) and presence (solid line) of pectin at 17 µM (**A**), glucose at 18 mM (**B**) and alginate at 11 µM (**C**). (**D**) Differential absorbance at 280 nm (∆Abs_280_) as a function of pectin concentration.

To determine the level of affinity of SPH1118 to pectin, UV absorption spectroscopy at 200–400 nm was conducted using SPH1118 in the absence or presence of pectin. This method is based on change in UV absorption accompanying ligand binding. In fact, UV absorption change was observed in SPH1118 in the presence of pectin (Fig. 5A) compared with that in its absence; however, no change occurred in the presence of glucose (Fig. 5B) and alginate (Fig. 5C) as ligands. These results indicate that SPH1118 specifically binds to pectin. Absorbance of SPH1118 at 280 nm was further measured at various concentrations of pectin. The results showed that absorbance increased with increasing pectin concentration. Changes in absorbance were then plotted against the pectin concentration (Fig. 5D). The resulting saturation curve indicated a dissociation constant (*K*_d_) of SPH1118 for pectin of 8.5 µM at the estimated molecular weight of pectin of 200,000^29^.

### Substrate specificity of SPH1118

As described in the Introduction, pectin comprises three domains^12^: PG, RG-I and RG-II. PG is the main component of pectin, and GalUA is linearly polymerised. RG-I shows a repeating structure of α-1,2 and α-1,4 bonds of l-rhamnose and GalUA as the main chain and side chains composed of three variations, namely arabinans comprising arabinose; galactans comprising galactose; and arabinogalactans comprising arabinose and galactose. Moreover, RG-I contains xylose and fucose. In RG-II, various monosaccharides are added as side chains to the main PG chain.

**Figure 6:**
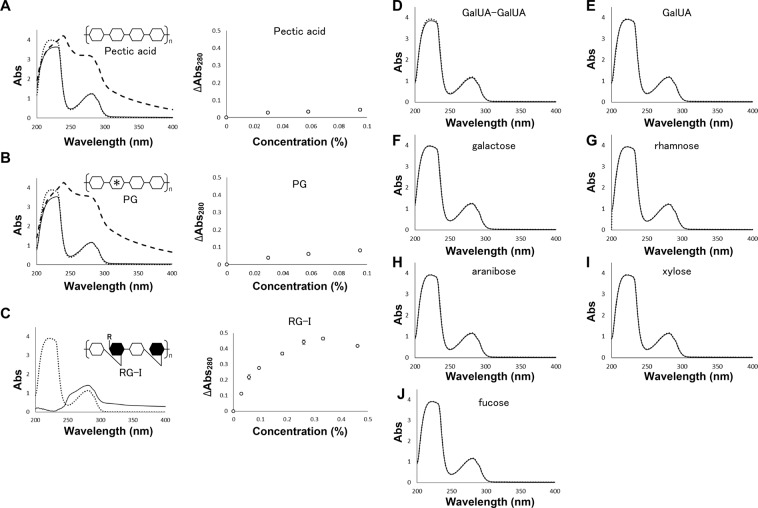
SPH1118 binding to RG-I of pectin. UV absorption spectrum of SPH1118 in the absence (dotted line) and presence of pectin-related compounds: pectic acid (**A**, left), PG (**B**, left), RG-I (**C**, left), GalUA–GalUA (**D**), GalUA (**E**), galactose (**F**), rhamnose (**G**), arabinose (**H**), xylose (**I**) and fucose (**J**). (**A**–**C**, left) solid line (0.1% each ligand), thick dotted line (0.2% each ligand). (**A**–**C**, right) ∆Abs_280_ as a function of ligand. (**D**–**J**) solid line (1.5 mM each ligand).

To analyse the substrate specificity of SPH1118 binding to pectin, the UV absorption spectrum at 200–400 nm was examined in the absence or presence of the following pectin components: pectic acid, PG, RG-I, digalactunoic acid (GalUA–GalUA), GalUA, galactose, rhamnose, arabinose, xylose and fucose. The addition of pectic acid, PG and RG-I to SPH1118 affected the UV spectrum (Fig. 6A–C, left), whereas that of GalUA–GalUA, GalUA, galactose, rhamnose, arabinose, xylose and fucose did not (Fig. 6D–J). Absorbance at 280 nm was monitored at various concentrations of pectic acid, PG and RG-I (Fig. 6A–C, right). In the presence of pectic acid and PG as ligands, the UV spectrum changed drastically when the substrate concentration increased from 0.1% to 0.2%, suggesting that SPH1118 was unfolded by the addition of excess amounts of these sugars. Because pectic acid and PG are acidic sugars, these substrates decreased the pH at concentrations higher than 0.2%. Absorbance of SPH1118 in the presence of RG-I changed in a ligand-concentration-dependent manner (Fig. 6C), indicating that SPH1118 binds to RG-I in pectin.

## Discussion

In the present study, SPH1118 was identified to be a pectin-binding protein acting as a trigger for the expression of chemotaxis towards the macromolecule pectin and its assimilation. Based on topological analysis using the PSORT program^30^, SPH1118 may be localised exclusively in the periplasm. Through homology analysis with a sequence identity of ~50%, some genes in the vicinity of *sph1118* are predicted to encode members of the ABC transporter system for the import of oligopeptides as follows: SPH1114, ATP-binding protein of the oligopeptide ABC transporter; SPH1115, permease of the oligopeptide ABC transporter; SPH1116, permease of the oligopeptide ABC transporter; SPH1117, solute-binding protein (fragmented) associating with the oligopeptide ABC transporter and SPH1118, a solute-binding protein associating with the oligopeptide ABC transporter. These topological and homology analyses suggested that the periplasmic solute-binding protein-dependent ABC transporter system functions as an oligopeptide importer in strain A1 cells. In fact, using DNA microarray analysis, *sph1114*–*sph1118* transcription has been demonstrated to be upregulated in strain A1 cells grown in a yeast extract medium containing oligopeptides compared with that in cells grown in an alginate minimal medium^31^. SPH1118 may bind to both oligopeptides and pectin, although further experiments on this interaction are required.

Strain *Δsph1118* cells showed decreased pectin assimilation compared with motile wild-type strain A1-MP cells, although they grew on pectin to some extent (Fig. S2). If SPH1118 is exclusively involved in pectin import, strain *Δsph1118* cells should completely lose their pectin-assimilating ability. This suggests the presence of another solute-binding protein for pectin import. In the plant pathogenic bacterium *D. dadantii*, TogMNAB is the ABC transporter system involved in pectin import^20^; therefore, a search for the TogMNAB-like system was undertaken in the whole-genome sequence of strain A1. The results revealed that SPH1155, SPH1156, SPH1157 and SPH1158 corresponded to TogM, TogN, TogA and TogB, respectively, with sequence identities of 38–57%, suggesting that SPH1155–SPH1158 function as other ABC transporters involved in pectin import. Particularly in strain *Δsph1118* cells, SPH1158 might act as another pectin-binding protein in the place of SPH1118. Distinct from *sph1118*-complemented strain *Δsph1118* cells, *sph1117*-*sph1118*-complemented strain A1-M5 cells showed no restoration of pectin assimilation. Among mutated genes in strain A1-M5 (Table 1), *sph1586*, *sph1588* and *sph3575* exhibited significant identity with pectin-degrading enzymes (pectate lyases). These mutations may lead to the decreased pectin assimilation by *sph1117*-*sph1118*-complemented strain A1-M5 cells.

In *D. dadantii*, TogB functions as a bifunctional protein responsible for chemotaxis towards pectin oligosaccharides as well as oligomer import^20^, although its chemotaxis towards the polysaccharide pectin remains unclear. TogB binds to pectin oligosaccharides including the disaccharide GalUA–GalUA^21^. Conversely, SPH1118 appeared to prefer polymers (specifically RG-I in pectin) over oligosaccharides and monosaccharides because the disaccharide remained inert as an SPH1118 substrate in our assay. This feature of SPH1118 confers strain A1-MP cells with the ability to exhibit chemotaxis towards the macromolecule pectin.

**Figure 7:**
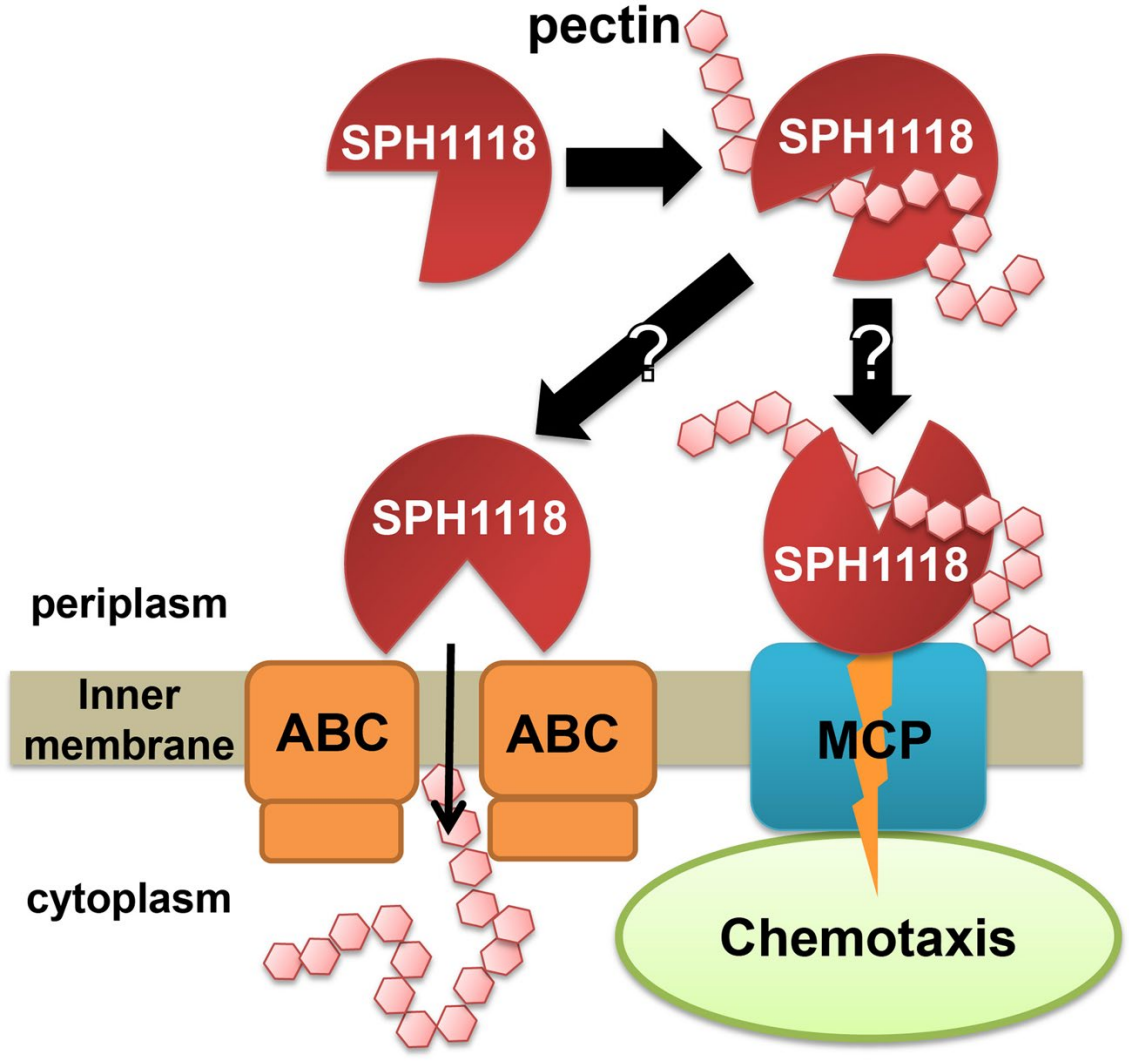
Strain A1 chemotaxis/import model towards pectin. Pectin-bound SPH1118 probably interacts with the MCP-like receptor for expressing chemotaxis towards pectin and with the ABC transporter to deliver the substrate for pectin assimilation.

Although the identification of pectin import and degradation pathways is required in strain A1 cells, a rough model of the physiological role of SPH1118 was postulated through this study (Fig. 7). Pectin and/or its fragmented forms are incorporated into the periplasm by candidate porin-like proteins such as KdgM^32^. Once SPH1118 binds to pectin in the periplasm, pectin-bound SPH1118 associates with the MCP-like receptor to exhibit chemotaxis towards pectin. Alternatively, pectin-bound SPH1118 delivers the substrate to the ABC transporter embedded in the inner membrane, and the substrate is assimilated for bacterial growth. In fact, the *K*_d_ value of SPH1118 for pectin was comparable to that of TogB for pectin oligosaccharide^21^ and MBP for maltose^33^. The overall scheme of alginate import, degradation and metabolism has been well characterised (Fig. S1). Similar to the present study, another study analysing the factors related to the exhibition of chemotaxis towards alginate through the recognition of polysaccharide in strain A1 cells is underway.

In conclusion, this is the first report on the identification of a protein triggering chemotaxis towards pectin and its assimilation through polysaccharide recognition and binding.

## Materials and Methods

### Materials

Sodium alginate (average molecular weight: 300,000) from *Eisenia bicyclis* and agar were purchased from Nacalai Tesque. Pectin from *Citrus* fruits, GalUA–GalUA and SYPRO Orange were purchased from Sigma-Aldrich. l-Rhamnose and GalUA were obtained from Wako. The pectin component RG-I was from purchased Megazyme and pectic acid from Lancaster. Restriction endonucleases and DNA-modifying enzymes were obtained from Toyobo. Oligonucleotides used in this study were synthesised by Hokkaido System Science (Table S2). All other chemicals used were analytical-grade and commercially available.

### Bacterial strains and culture

Strains A1 (non-motile wild-type), A1-M5 (motile with chemotaxis towards alginate but not pectin), strain A1-MP (motile with chemotaxis towards alginate and pectin) and their derivatives were used. To investigate polysaccharide assimilation ability, bacterial cells were aerobically cultured at 30 °C in an alginate or a pectin minimal medium [0.5% (w/v) alginate or pectin, 0.1% (NH_4_)_2_SO_4_, 0.1% KH_2_PO_4_, 0.1% Na_2_HPO_4_ and 0.01% MgSO_4_·7H_2_O (pH 7.2)]. To evaluate chemotactic response, bacterial cells were aerobically cultured at 30 °C in an alginate/yeast extract medium [0.5% alginate, 0.1% (NH_4_)_2_SO_4_, 0.1% KH_2_PO_4_, 0.1% Na_2_HPO_4_, 0.5% yeast extract and 0.01% MgSO_4_·7H_2_O (pH 7.2)]. Antibiotics were appropriately added at the following concentrations: sodium ampicillin, 100 µg/ml; kanamycin sulphate, 25 µg/ml; tetracycline hydrochloride, 10 µg/ml and chloramphenicol, 10 µg/ml. For DNA manipulation and protein expression, *E. coli* cells were aerobically cultured at 37 °C in LB medium [1% tryptone, 0.5% yeast extract and 1% NaCl (pH 7.2)]^34^.

### Chemotaxis assay

Chemotaxis assay was conducted as reported previously, with slight modifications^35^. At the centre of the chemotaxis assay plates [0.1% (NH_4_)_2_SO_4_, 0.1% KH_2_PO_4_, 0.1% Na_2_HPO_4_, 0.01% MgSO_4_·7H_2_O and 0.25% agar (pH 7.2)], 10 µl of 2% polysaccharide solution was spotted linearly at regular intervals. The plates were incubated at 4 °C for 16 h to create a concentration gradient. The bacterial cells were then spotted around the polysaccharide spots and incubated at 30 °C for 5 days to investigate cell chemotaxis towards polysaccharides (Fig. 1A).

### Whole-genome sequencing

Genomic DNA of strain A1-M5 cells was extracted using NucleoSpin Tissue (MACHEREY-NAGEL) and subjected to next-generation sequencing (Hokkaido System Science). The determined genome sequence of strain A1-M5 was compared with that of the wild-type strain A1 to identify mutated genes in the former strain.

### Gene disruption

Plasmids for the disruption of strain A1 genes [*sph####* (####, digit number)] were constructed as follows. DNA fragments containing the *sph####* gene were amplified by polymerase chain reaction (PCR) using KOD-Plus-Neo (Toyobo) using genomic DNA of strain A1 as a template and two synthetic oligonucleotides as primers (sph#### f and sph#### r). All primers used in this study are listed in Table S2. Fragments amplified by PCR were isolated and ligated using Ligation High (Toyobo) into *Hin*cII-digested pUC119 (Takara Bio). The resulting plasmids were designated as pUC119-sph####. To obtain the fragmented *sph####* split in the middle, linear DNA was amplified using pUC119-sph#### as a template and two synthetic oligonucleotide primers (sph#### f inverse and sph#### r inverse). The Km^r^ gene was amplified by PCR using pUC4K as a template and two synthetic oligonucleotide primers (Km-F and Km-R). Km^r^ was inserted in the middle of *sph####* in the pUC119-sph#### plasmid by ligation. The resultant plasmids with a disrupted *sph####* were designated pUC119-sph####::Km^r^. sph####::Km^r^ genes were amplified by PCR using pUC119-sph####::Km^r^ as a template and two synthetic oligonucleotide primers (sph#### f and sph#### r). The PCR products were ligated with *Hin*cII-digested pKTY320^36^. The resultant plasmids were designated as pKTY320-sph####::Km^r^. Each plasmid was introduced into *E. coli* strain DH5α cells. *E. coli* cells harbouring pKTY320-sph####::Km^r^ were used to transconjugate strain A1 and its derivative cells through triparental mating^37^ in the presence of *E. coli* strain HB101 cells harbouring pRK2013^38^ as a helper. The *sph####* gene disruptant was screened on the basis of its resistance or sensitivity to antibiotics. Gene disruption was confirmed by DNA sequencing of the gene of interest.

### Isolation of strain A1-M5 derivatives with chemotaxis towards pectin

A genomic DNA library of strain A1 using a broad-host range vector (pKS13) containing the tetracycline-resistant gene has been constructed in *E. coli* strain DH5α cells^16^. This genomic library of strain A1 was introduced into strain A1-M5 cells through triparental mating. The transformed strain A1-M5 cells were spotted onto a chemotaxis assay plate, with pectin spotted at the centre. After incubation at 30 °C for 5 days, the bacterial cells moving towards pectin were collected and seeded on an alginate minimal medium plate containing 1.5% agar and 10 µg/ml tetracycline hydrochloride. The plasmids containing strain A1 genomic DNA fragments were isolated from the transformed strain A1-M5 cells showing chemotaxis towards pectin, and DNA sequences of the genomic fragments in the plasmids were analysed using two synthetic oligonucleotide primers (pKS_F1 and pKS_R2).

### Gene complementation

Plasmids for *sph####* gene complementation were constructed as follows. DNA fragments containing *sph####* were amplified by PCR using the genomic DNA of strain A1 as a template and two synthetic oligonucleotides as primers (sph#### BamHI in-fusion f and sph#### BamHI in-fusion r). Fragments amplified by PCR were cloned into *Bam*HI-digested pKS13 using the In-Fusion HD cloning kit (Clontech), and the resultant plasmids were designated pKS13-sph####. These plasmids were introduced into *E. coli* strain DH5α cells. The resultant transformed *E. coli* cells were used to transconjugate strain A1 gene disruptant cells through triparental mating. The gene-complemented cells were screened on the basis of their resistance to antibiotics. Gene complementation was confirmed by DNA sequencing.

### SPH1118 overexpression and purification

The strain A1 gene *sph1118* was amplified by PCR using KOD-Plus-Neo and two primers (sph1118-NdeI in-fusion and sph1118-XhoI in-fusion) from strain A1-MP cells. The amplified gene without a stop codon was purified using the Gel/PCR DNA Isolation System (VIOGENE) and inserted into *Nde*I/*Xho*I-digested pET-21b plasmid (Novagen) via in-fusion reaction at 50 °C for 15 min using 5× In-fusion HD Enzyme Premix (Clontech). Correctness of the *sph1118* sequence in the resultant plasmid pET21b-sph1118 was confirmed by DNA sequencing. *E. coli* strain BL21(DE3) cells were transformed with pET21b-sph1118 to express recombinant SPH1118 containing a His tag at the C-terminus.

Unless otherwise specified, all procedures were conducted at 4 °C. The transformant *E. coli* strain BL21(DE3)/pET21b-sph1118 cells were aerobically grown at 30 °C in 1.5 l of LB medium containing 100 μg/ml sodium ampicillin. At the exponential growth phase, isopropyl β-d-thiogalactopyranoside was added to the culture at a final concentration of 0.1 mM. The bacterial cells were further grown overnight, collected by centrifugation at 14,450 × g for 5 min and resuspended in 20 mM Tris–HCl (pH 8.0). Cells were lysed using ultrasonication at 9 kHz for 20 min followed by centrifugation at 18,000 × g for 20 min to obtain supernatant as a cell extract. The cell extract was added to 15 ml TALON resin (Clontech) equilibrated with 20 mM Tris–HCl (pH 8.0), 0.5 M NaCl and 10 mM imidazole. After washing with the same buffer, proteins bound to the resin were eluted by increasing the concentration of imidazole in the buffer to 200 mM. Absorbance at 280 nm was monitored during elution. After elution, proteins were subjected to SDS–PAGE. Fractions containing SPH1118 were collected, concentrated and applied to a HiLoad 16/60 Superdex 200-pg column equilibrated with 20 mM Tris–HCl (pH 8.0) containing 150 mM NaCl. Proteins were eluted with the same buffer using a 2-ml fraction collected every 2 min. Protein fractions were subjected to SDS–PAGE, and fractions containing SPH1118 were dialysed using 20 mM Tris–HCl (pH 8.0) for 5 h. The dialysate was used as the purified SPH1118. Protein concentration of SPH1118 was determined using a molar extinction coefficient of 122,730 (M^−1^ cm^−1^) calculated using the *ExPASy ProtParam* tool^39^.

### Protein–ligand interaction measurement

Interactions between SPH1118 and each ligand were analysed with two methods: DSF^28^ and UV absorption spectroscopy. Thermal stability of the protein in the absence or presence of pectin was measured by DSF. A dye, SYPRO Orange, was used to monitor protein unfolding because this dye hydrophobically binds to the inside of the protein and shows high fluorescence intensity according to binding to the unfolded proteins. Proteins (0.15 mg/ml) were mixed with 60-fold diluted SYPRO Orange in 20 mM Tris–HCl (pH 8.0) in the absence or presence of pectin. Fluorescence intensity of the dye was monitored as a function of temperature to determine *T*_m_ values of the proteins.

To calculate the *K*_d_ values of SPH1118 towards ligands, UV absorption spectra with elevated concentrations of ligands were measured in 20 mM Tris–HCl (pH 8.0) at 30 °C using a Shimadzu UV-2600 spectrophotometer. Specifically, absorbance at 280 nm was plotted as a function of ligand concentration to calculate the *K*_d_ values by fitting to a Michaelis–Menten curve. Interactions of SPH1118 with mono/oligo/polysaccharides were evaluated using UV absorption spectroscopy.

## Supplementary information

Supplementary Information.

Table S1

## Data Availability

Genome sequence of strain A1-M5 is deposited in the GenBank, EMBL and DDBJ databases under accession number DRA006486.
