# Supplementary material for: Bacterial chemotaxis towards polysaccharide pectin by pectin-binding protein
Source: Sci Rep. 2020 Mar 4;10:3977. doi: 10.1038/s41598-020-60274-1 (PMC7055323; doi:10.1038/s41598-020-60274-1)
Supplement: Supplementary file 1 — Supplementary Information. [file 41598_2020_60274_MOESM1_ESM.pdf]

**Supplementary Information**

# Bacterial chemotaxis towards polysaccharide pectin by pectin-binding protein

Hidenori Konishi[1], Mamoru Hio[2], Masahiro Kobayashi[1], Ryuichi Takase[1,2], & Wataru Hashimoto[1,2]

[1]Laboratory of Basic and Applied Molecular Biotechnology, Division of Food Science and Biotechnology, Graduate School of Agriculture, Kyoto University, Uji, Kyoto 611-0011, Japan; [2]Laboratory of Basic and Applied Molecular Biotechnology, Department of Food Science and Biotechnology, Faculty of Agriculture, Kyoto University, Uji, Kyoto 611-0011, Japan; Correspondence and requests for materials should be addressed to W. H. (email: whasimot@kais.kyoto-u.ac.jp)

**Table S1.** Mutations in the genome of strain A1-M5.

An excel file for Table S1 was separately uploaded.

**Table S2.** Primers used in this study.

| Primer | Sequence (5'-3') |
|---|---|
| pKS_F1 | AAAAATGGCAGCGCTGGCAGTCCTT |
| pKS_R2 | GGCGGTGCTACAGAGTTCTTGAAGT |
| r-3kbp→6kbp f | TGATCGACCTGCAAGAGCGCATCCA |
| r-3kbp→6kbp r | TGTTCAAGCGCTCGACGCGAGTGCT |
| f→3.2kbp | GGGTCGTCGTCAAAGCGTCCTGTCA |
| r-6kbp→9kbp f | ACACACCCAAAGCGCCGCTTGACTG |
| r-6kbp→9kbp r | GCTCAGGTCAACATGAGCAACCTGC |
| f-3.2kbp→6.2kbp f | TGCTCAAGCCACGTTTGGCCGCGTT |
| f-3.2kbp→6.2kbp r | CACGACGCGCCCAATGTTCACCTCA |
| r-9kbp→12kbp f | GGTCGTAGCTGTCGTCTCAGTCATT |
| r-9kbp→12kbp r | TTTCACGCAAGCGCCGATGCTGGAA |
| f-6.2kbp→9.2kbp f | ATGAGGTGAACATTGGGCGCGTCGT |
| f-6.2kbp→9.2kbp r | ATGGCGATGTCCAGCCCCACGGTAT |
| r-12kbp→ f | TTGCGTGAAAGCAGCAGCGATCGCC |
| f-9.2kbp→12kbp f | ATACCGTGGGGCTGGACATCGCCAT |
| f-9.2kbp→12kbp f | GACCAACCACGTCAGCGGTGCGATA |
| f-12kbp→f | TATCGCACCGCTGACGTGGTTGGTC |
| r-1289838~ | TAGACCAGCGTCTGCAATTCCAGGC |
| f-3135268~ | TACGCTGTGGGAGACCGACGAGGTT |
| sph2942 BamHI infusion f | TCGCGCGACCGGATCAATGCAAGGCCGTTCAACGC |
| sph2942 BamHI infusion r | AACGTTGCCCGGATCCGACAAGGGCAGATACACGT |
| sph1119 BamHI infusion f | TCGCGCGACCGGATCGCGGGAACATTTCCTTGTTC |
| sph1119 BamHI infusion r | AACGTTGCCCGGATCGTCGATTACGCAACCAGCCG |
| sph1117 BamHI infusion f | TCGCGCGACCGGATCCCGTCGTACCACCGTTAACC |
| sph1117 BamHI infusion r | AACGTTGCCCGGATCGTATCGGAGAAGTTCGCCCG |
| sph1118 BamHI infusion f | TCGCGCGACCGGATCACGAACTTGCCGATCGACCA |
| sph1118 BamHI infusion r | AACGTTGCCCGGATCATTGGCGCCCATACCTCCAG |
| sph2733 BamHI infusion f | TCGCGCGACCGGATCTTCAAGCTGGGCTACCTCGC |
| sph2733 BamHI infusion r | AACGTTGCCCGGATCGGACGTACGGATGATGTCGA |
| sph1118 BamHI infusion f new | TCGCGCGACCGGATCTGATGATCACGGGGATCACC |
| sph1118 BamHI infusion r new | AACGTTGCCCGGATCTTAAAACGGCCGCGCAACCA |
| sph1117 f | AAGCGATACCACATCAGCGTCCACT |
| sph1117 r | AGAAGCGTATGGCGCTGTACGACCA |
| sph1118 f | TACGCTTCTTCATGCTTTCGTTCGG |
| sph1118 r | TCGTAGTTCATCCCTAGGAGGACAA |
| sph1119 f | ATGAGTCGACCCCTTCATTTTCTGC |
| sph1119 r | TCAGGCGTTTGAACTACCTGATGTC |
| sph1117 f inverse | AAGTCATCGGCACGGTTCGTCCGGC |
| sph1117 r inverse | TGAGTCTTGGCGGCGACGAACTGGT |
| sph1118 f inverse | GACGGTGTAATCGTCAACCTTCGAA |
| sph1118 r inverse | TGGCTATGGACCGTGACGAGATCAA |
| sph1119 f inverse | GGGCGACCTCCAGTGCCTCGGTATT |
| sph1119 r inverse | AATGACCGCGCACGCCATGCACGGA |
| SPH1118 NdeI（S） | GGCATATGGCTGCTTTCACGCAAGCGCCGA |
| SPH1118 NdeI（L） | GGCATATGATGAGGTTTTACTCTCGCAAGTTTGCG |
| Km-F | GGGGGCGCTGAGGTCTGCCTCGTGAAG |
| Km-R | GGGGGAAAGCCACGTTGTGTCTCAAAA |
| sph1118-NdeI in fusion | AAGGAGATATACATATGGCTGCTTTCACGCAAGCGCCGA |
| sph1118-XhoI in fusion | GGTGGTGGTGCTCGAGCTTGGAGAAGTACCACTGCT |

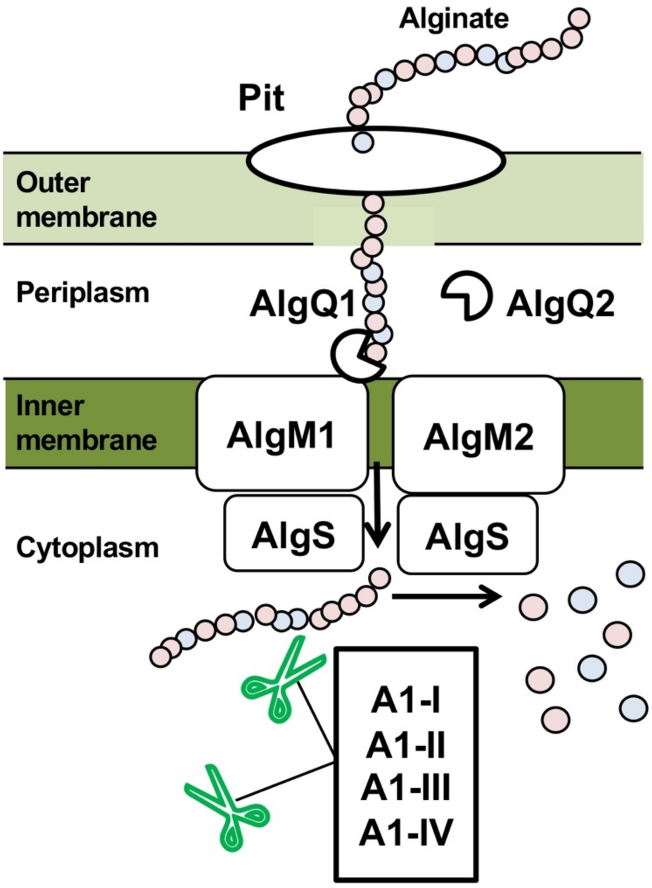


**Figure S1.** Import and degradation of alginate polysaccharide by strain A1 cells. Extracellular alginate is incorporated to the periplasm through the cell-surface pit and bound to the periplasmic binding protein (AlgQ1 or AlgQ2). The binding protein delivers the polysaccharide to the ABC transporter (AlgM1-AlgM2/AlgS-AlgS), which imports alginate to the cytoplasm with an energy from ATP hydrolysis by AlgS-AlgS. Alginate is degraded to constituent monosaccharides by three endotype (A1-I, II, and III) and a exotype (A1-IV) alginate lyases.

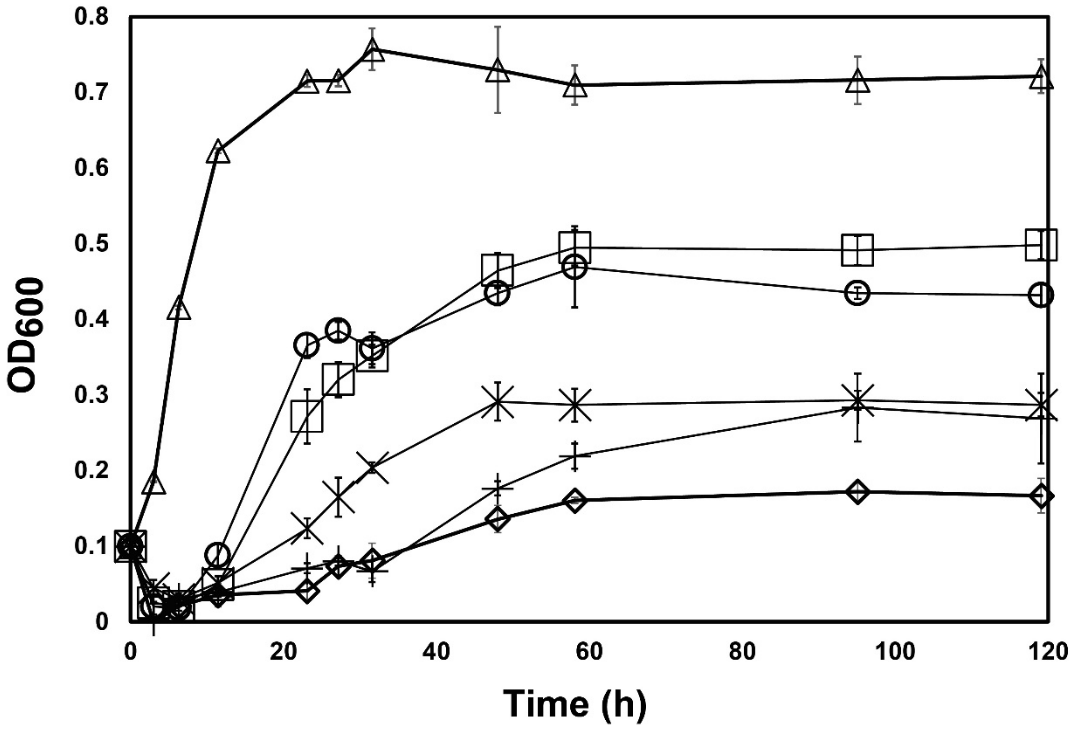


**Figure S2.** Assimilation of pectin by strain A1 cells. Bacterial cells were cultured in test tubes (*n* = 3). Values indicate the average optical density at 600 nm ($OD_{600}$) as follows: triangle, non-motile wild-type strain A1 cells; circle, strain A1-MP cells; rhombus, strain A1-M5 cells ; square, *sph1118*-complemented strain *Δsph1118* cells derived from strain A1-MP; cross, strain *Δsph1118* cells derived from strain A1-MP; plus, *sph1117-sph1118*-complemented A1-M5 cells. Error bars indicate standard deviations.

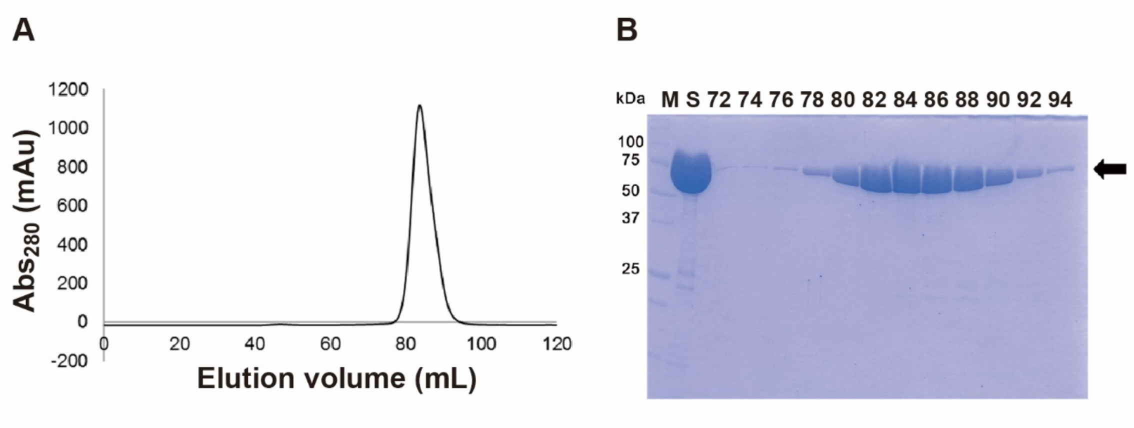


**Figure S3.** Purification of SPH1118. (**A**) Elution profile of the recombinant SPH1118 from gel filtration column chromatography. (**B**) SDS-PAGE profile of the recombinant SPH1118. Lane S, proteins before subjected to the gel filtration column chromatography; lanes 72-94, fractions cotaining the eluted protein from the chromatography. This electrophoretic profile is not an image cropped from different parts of the same gel or from different gels.
